# Supplementary material for: Recurrent Plasmodium falciparum parasitemia and drug resistance mutations during intermittent preventive treatment of malaria in pregnancy in Uganda
Source: medRxiv. 2025 Oct 20:2025.10.18.25338277. Preprint. [Version 1] doi: 10.1101/2025.10.18.25338277 (PMC12633580; doi:10.1101/2025.10.18.25338277)
Supplement: Supplement 1 [file NIHPP2025.10.18.25338277v1-supplement-1.pdf]

Supplementary Table 1. Risk of recurrent parasitemia following administration of different IPTp regimens stratified by gravidity

IPTp regimen	Gravidity[1]	All infections			Recrudescent infections		
		Cumulative risk of recurrent parasitemia (95% CI)	HR (95% CI)	p-value	Cumulative risk of recurrent parasitemia (95% CI)	HR (95% CI)	p-value
Any parasitemia during 28-day follow-up							
SP	Multigravida (n=227)	32.7% (26.8-39.4%)	Reference group		24.4% (19.1-30.8%)	Reference group	
	Secundigravida (n=195)	47.6% (40.4-55.2%)	1.48 (1.10-2.00)	<0.001	29.8% (23.5-37.3%)	1.21 (0.82-1.80)	0.33
	Primigravida (n=390)	76.9% (72.5-81.0%)	2.68 (2.10-3.42)	<0.001	54.6% (49.5-59.9%)	2.43 (1.79-3.29)	<0.001
DP	Multigravida (n=79)	2.8% (0.7-10.8%)	Reference group		1.3% (0.2-8.7%)	Reference group	
	Secundigravida (n=69)	9.3% (4.3-20.0%)	3.37 (0.58-19.5)	0.17	4.7% (1.5-14.0%)	3.41 (0.23-51.6)	0.38
	Primigravida (n=121)	1.8% (0.5-7.2%)	0.63 (0.09-4.37)	0.64	0.8% (0.1-5.7%)	0.64 (0.004-10.3)	0.75
DP+SP	Multigravida (n=88)	1.1% (0.2-7.8%)	Reference group		1.1% (0.2-7.8%)	Reference group	
	Secundigravida (n=60)	8.9% (3.4-22.0%)	5.87 (0.69-49.7)	0.10	0%	NA	
	Primigravida (n=148)	3.6% (1.4-9.2%)	2.36 (0.27-20.4)	0.41	0.9% (0.1-6.2%)	0.59 (0.04-9.14)	0.71
Parasitemia during 28-day follow-up with symptoms requiring antimalarial therapy							
SP	Multigravida (n=227)	4.1% (2.2-7.8%	Reference group		3.2% (1.5-6.6%)	Reference group	
	Secundigravida (n=195)	7.5% (4.5-12.4%)	1.82 (0.81-4.12)	0.15	4.8% (2.5-9.1%)	1.50 (0.57-4.00)	0.41
	Primigravida (n=390)	13.1% (10.1-17.0%)	3.39 (1.66-6.92)	0.001	10.0% (7.4-13.5%)	3.28 (1.45-7.44)	0.004
DP	Multigravida (n=79)	1.3% (0.2-8.7%)	Reference group		1.3% (0.2-8.7%)	Reference group	
	Secundigravida (n=69)	2.9% (0.7-11.2%)	2.33 (0.22-24.6)	0.48	1.5% (0.2-9.8%)	1.16 (0.08-17.6)	0.91
	Primigravida (n=121)	0%	NA		0%	NA	
DP+SP	Multigravida (n=88)	1.1% (0.2-7.8%)	Reference group		1.1% (0.2-7.8%)	Reference group	
	Secundigravida (n=60)	0%	NA		0%	NA	
	Primigravida (n=148)	0%	NA		0%	NA	

[1]Multigravidae include secundigravidae and those with >2 pregnancies.

medRxiv preprint doi: https://doi.org/10.1101/2025.10.18.25338277; this version posted October 20, 2025. The copyright holder for this preprint (which was not certified by peer review) is the author/funder, who has granted medRxiv a license to display the preprint in perpetuity.
It is made available under a CC-BY 4.0 International license .

Supplementary Table 2. Selection of molecular markers associated with high-grade SP resistance only including pure mutant samples

IPTp regimen	Number of prior doses	DHFR I164L			DHPS A581G			DHFR I164L and/or DHPS A581G		
		Prevalence	RR (95% CI)*	p-value	Prevalence	RR (95% CI)*	p-value	Prevalence	RR (95% CI)*	p-value
SP	0	7/253 (2.7%)	Reference group		2/253 (0.8%)	Reference group		9/253 (3.6%)	Reference group	
	1-3	41/416 (9.9%)	3.52 (1.67-7.41)	0.001	4/416 (1.0%)	1.26 (0.27-5.79)	0.77	43/416 (10.3%)	2.96 (1.54-5.68)	0.001
	4-6	26/143 (18.2%)	6.32 (2.98-13.4)	<0.001	6/143 (4.2%)	5.19 (1.18-22.9)	0.03	31/143 (21.7%)	5.86 (3.04-11.3)	<0.001
	Any (1-6)	67/559 (12.0%)	4.17 (2.04-8.51)	<0.001	10/559 (1.8%)	2.29 (0.57-9.24)	0.24	74/559 (13.2%)	3.65 (1.96-6.78)	<0.001

medRxiv preprint doi: https://doi.org/10.1101/2025.10.18.25338277; this version posted October 20, 2025. The copyright holder for this preprint (which was not certified by peer review) is the author/funder, who has granted medRxiv a license to display the preprint in perpetuity.
It is made available under a CC-BY 4.0 International license .

Supplementary Table 3. Selection of molecular markers associated with high-grade DP resistance[1]

IPTp regimen	Number of prior doses	PfCRT K76T			PfMDR-1 N86Y			PfK13 C469Y or A675V		
		Prevalence	RR (95% CI)*	p-value	Prevalence	RR (95% CI)*	p-value	Prevalence	RR (95% CI)*	p-value
SP	0	2/253 (0.8%)	Reference group		0/252 (0%)	Reference group		6/253 (2.4%)	Reference group	
	1-3	1/416 (0.2%)	0.18 (0.03-1.18)	0.07	0/416 (0%)	NA		16/416 (3.9%)	1.53 (0.65-3.61)	0.33
	4-6	1/143 (0.7%)	1.43 (0.17-12.1)	0.74	0/143 (0%)	NA		4/143 (2.8%)	1.09 (0.31-3.87)	0.89
	Any (1-6)	2/559 (0.4%)	0.42 (0.07-2.73)	0.37	0/559(0%)	NA		20/559 (3.6%)	1.42 (0.60-3.36)	0.42
DP	0	3/255 (1.2%)	Reference group		0/254 (0%)	Reference group		10/254 (3.9%)	Reference group	
	1-3	1/9 (11.1%)	13.3 (2.55-69.7)	0.002	0/9 (0%)	NA		0/9 (0%)	NA	
	4-6	0/5 (0%)	NA		0/5 (0%)	NA		0/5 (0%)	NA	
	Any (1-6)	1/14 (7.1%)	7.28 (1.01-52.7)	0.05	0/14 (0%)	NA		0/14 (0%)	NA	
DP+SP	0	2/263 (0.8%)	Reference group		1/263 (0.4%)	Reference group		12/263 (4.6%)	Reference group	
	1-3	0/18 (0%)	NA		0/18 (0%)	NA		2/18 (11.1%)	2.48 (0.62-9.84)	0.20
	4-6	0/15 (0%)	NA		0/15 (0%)	NA		1/15 (6.7%)	1.52 (0.23-9.81)	0.66
	Any (1-6)	0/33 (0%)	NA		0/33 (0%)	NA		3/33 (9.1%)	2.06 (0.64-6.65)	0.23

[1]Adjusted for repeated measures in the same study participant

medRxiv preprint doi: https://doi.org/10.1101/2025.10.18.25338277; this version posted October 20, 2025. The copyright holder for this preprint (which was not certified by peer review) is the author/funder, who has granted medRxiv a license to display the preprint in perpetuity.
It is made available under a CC-BY 4.0 International license .
